# Study protocol: rehabilitation including social and physical activity and education in children and teenagers with cancer (RESPECT)

**DOI:** 10.1186/1471-2407-13-544

**Published:** 2013-11-14

**Authors:** Troels Thorsteinsson, Anne Sofie Helms, Lis Adamsen, Lars Bo Andersen, Karen Vitting Andersen, Karl Bang Christensen, Henrik Hasle, Carsten Heilmann, Nete Hejgaard, Christoffer Johansen, Marianne Madsen, Svend Aage Madsen, Venka Simovska, Birgit Strange, Lone Friis Thing, Peder Skov Wehner, Kjeld Schmiegelow, Hanne Baekgaard Larsen

**Affiliations:** 1 Department of Pediatrics and Adolescent Medicine, Copenhagen University Hospital Rigshospitalet, Blegdamsvej 9, 2100 Copenhagen, Denmark; 2 Faculty of Health Sciences, The University of Copenhagen, Blegdamsvej 2b, 2100 Copenhagen, Denmark; 3 Department of Public Health and Faculty of Health Sciences, The University Hospitals Centre for Nursing and Care Research (UCSF), Copenhagen University Hospital Rigshospitalet, Ryesgade 27, 2200 Copenhagen, Denmark; 4 Institute for Sport Sciences and Clinical Biomechanics, The University of Southern Denmark, Campusvej 55, 5230 Odense M, Denmark; 5 Department of Biostatistics, The University of Copenhagen, Øster Farimagsgade 5, P.O. Box 2099, , 1014 Copenhagen, Denmark; 6 Department of Pediatrics, Aarhus University Hospital, Skejby, Brendstrupgårdsvej 100, 8200 arhus N, Denmark; 7 Øster Farimagsgade Skole, Øster Farimagsgade 41, 2100 Copenhagen Ø, Denmark; 8 Department of Oncology, Copenhagen University Hospital Rigshospitalet, Blegdamsvej 9, 2100 Copenhagen Ø, Denmark; 9 Head Survivorship Department, The Danish Cancer Society Research Center, Strandboulevarden 49, 2100 Copenhagen, Denmark; 10 Department of Education, Aarhus University, Campus Emdrup, Tuborgvej 164, 2400 Copenhagen, Denmark; 11 Department of Occupational Therapy and Physiotherapy, Copenhagen University Hospital Rigshospitalet, Blegdamsvej 9, 2100 Copenhagen, Denmark; 12 Department of Nutrition, Exercise and Sports, The University of Copenhagen, Nørre Allé 51-55, 2200 Copenhagen N, Denmark; 13 Department of Pediatric Hematology and Oncology, The H. C. Andersen Children’s Hospital, Odense University Hospital, Sdr. Boulevard 29, 5000 Odense C, Denmark

**Keywords:** Cancer, Pediatric, Children, Rehabilitation, Physical activity, Quality of life, Intervention, Peers, Controlled, School reentry

## Abstract

**Background:**

During cancer treatment children have reduced contact with their social network of friends, and have limited participation in education, sports, and leisure activities. During and following cancer treatment, children describe school related problems, reduced physical fitness, and problems related to interaction with peers.

**Methods/design:**

The RESPECT study is a nationwide population-based prospective, controlled, mixed-methods intervention study looking at children aged 6-18 years newly diagnosed with cancer in eastern Denmark (n = 120) and a matched control group in western Denmark (n = 120). RESPECT includes Danish-speaking children diagnosed with cancer and treated at pediatric oncology units in Denmark. Primary endpoints are the level of educational achievement one year after the cessation of first-line cancer therapy, and the value of VO_2max_ one year after the cessation of first-line cancer therapy. Secondary endpoints are quality of life measured by validated questionnaires and interviews, and physical performance. RESPECT includes a multimodal intervention program, including ambassador-facilitated educational, physical, and social interventions. The educational intervention includes an educational program aimed at the child with cancer, the child’s schoolteachers and classmates, and the child’s parents. Children with cancer will each have two ambassadors assigned from their class. The ambassadors visit the child with cancer at the hospital at alternating 2-week intervals and participate in the intervention program. The physical and social intervention examines the effect of early, structured, individualized, and continuous physical activity from diagnosis throughout the treatment period. The patients are tested at diagnosis, at 3 and 6 months after diagnosis, and one year after the cessation of treatment. The study is powered to quantify the impact of the combined educational, physical, and social intervention programs.

**Discussion:**

RESPECT is the first population-based study to examine the effect of early rehabilitation for children with cancer, and to use healthy classmates as ambassadors to facilitate the normalization of social life in the hospital. For children with cancer, RESPECT contributes to expanding knowledge on rehabilitation that can also facilitate rehabilitation of other children undergoing hospitalization for long-term illness.

**Trial registration:**

Clinical Trials.gov: file. NCT01772849 and NCT01772862

## Background

Each year 200 children and adolescents in Denmark are diagnosed with cancer. Over recent decades the increased understanding of cancer biology, improved surgery and chemotherapy, and generally intensified treatment, have resulted in 5-year survival rates above 80% [1-3]. However, the disadvantages of this improvement include severe acute and late effects [2-4], often involving isolation for long periods of time, both when in hospital and at home. This isolation reduces participation in activities with peers at school and in sporting activities [1,5,6]. As a result, important social interactions and the natural development of social skills with classmates are disrupted [5,6]. Following diagnosis, children are absent from school for an average of 85 days [7] during the first 12 to 18 months, and 3 years after diagnosis their school attendance is still irregular and many fail classes [8,9]. Childhood and adolescent cancer survivors report being bullied, feeling isolated [10-13], and having few or no friends [14-16]. Although register-based data show that they achieve the expected educational level post-treatment [17], such data do not address social and physical functioning [18-20]. Attending school may help provide normality, continuity, and security, in an abnormal life situation [21]. Few intervention studies have addressed children’s reentry into school during and following treatment. Being able to participate in normal school activities with peers includes being physically active. However, very few studies have been published on physical activity in children with cancer [22-24]. These studies have in general been burdened by the diversity of exercise and outcome measurements, and/or limited duration of the interventions [22,24-26]. Overall, the published studies have shown that children with cancer are less physically active and have decreased muscle strength, balance, and cardiovascular condition compared with peers, both during and following treatment [8,16,26,27]. The few studies carried out during treatment show that it is possible to improve children’s physical functioning, both during and following treatment [23].

The burden of the disease, and body modifications resulting from treatment and reduced physical activity, may lead to lower self-esteem and emotional well-being, and compromised social relationships, which negatively influence the quality of life for children with cancer [12,13,28,29]. However, it is unclear to what extent this is related to the disease and treatment burden, the child’s learning difficulties, physical decline, or changes in social position [30-32].

Importantly, none of the intervention studies address the potential effects of early rehabilitation from the time of diagnosis as a tool to maintain the children’s social network during treatment [28,30,32]. The Rehabilitation including Social and Physical activity and Education for Children and Teenagers with Cancer (RESPECT) study is inspired by Erving Goffman’s symbolic interaction theory [33-35], Thomas Scheff’s theory and concepts of emotional and social bonds [36,37], and Venka Simovska’s definition of interactive processes and empowerment [38].

The overall purpose of the RESPECT study is to examine whether involving healthy classmates at the hospital from the time of diagnosis and throughout treatment will improve the educational, physical, and social performance of children with cancer and facilitate their reentry into everyday life following treatment.

## Methods

### Trial design

RESPECT is a multimodal intervention program for children undergoing cancer treatment. This study is an integrated part of a newly established comprehensive rehabilitation program (CIRE) for children and adults during and following their cancer diagnosis. The overall aim of the CIRE program is to identify rehabilitation needs, apply early physical training, and to combine quantitative and qualitative research methods to understand the functional, cognitive, emotional, social, and physiological mechanisms involved in successful rehabilitation.

### Participants

Children aged 6-18 years diagnosed with cancer and treated with chemotherapy/irradiation, or diagnosed with Langerhans cell histiocytosis (LCH) or myelodysplastic syndrome (MDS) and treated with chemotherapy, at any pediatric oncology unit in Denmark are eligible for the study. All participants are enrolled at school at the time of diagnosis and are able to communicate in Danish. Children with mental disability (e.g. Down syndrome), severe co-morbidity, or terminal illness at the time of diagnosis are excluded. Patients diagnosed in the period 2013-2015 at the Copenhagen University Hospital Rigshospitalet (n = 120), are assigned to the intervention group.

Four control groups are identified: 1) children with cancer diagnosed at Odense University Hospital, Aarhus University Hospital, and Aalborg University Hospital in the period 2013-2015 (n = 120). Secondary control groups are 2) the sibling closest in age (regardless of gender) to the child with cancer, and 3) the child’s classmates. In addition, we include 4) a historical nationwide control group of children with cancer treated from February to December 2012 (n = 113 families).

### Interventions

#### ***Educational intervention***

The educational intervention includes a 90-minute presentation on cancer given at the child’s school and aimed at the child’s classmates. The information included covers the etiology of childhood cancer, the specific subtype of cancer, the treatment, the expected side effects, supportive care, everyday life at the hospital, communication and emotional strains, the importance of physical activity during treatment, and the role of the ambassadors. The teacher develops a weekly-updated curriculum, which the child is to follow in cooperation with the hospital schoolteachers and the ambassadors. At the meeting a consent form to be completed at home is handed out, on which all parents indicate whether their child is capable of acting as an ambassador for the classmate with cancer. In collaboration with the class teacher, the children with cancer, the families of the classmates, and the RESPECT research team, two ambassadors are selected. In this way the children with cancer each have two ambassadors assigned from their class. The ambassadors alternately visit the child with cancer when at hospital, at least twice monthly, throughout the treatment period. The ambassadors visit the pediatric oncology ward involved in the patient’s treatment and participate in the hospital school program, share meals in the kitchen, and participate in physical and social activities. The ambassadors therefore act as a bridge between the child’s everyday life at home and at the hospital, and serve as role models. Furthermore, the ambassador provides moral support, familiarity, and encouragement, and helps to create a friendly educational, physical, and social environment for the child with cancer. By involving healthy children, we will examine whether the creation of a more normal everyday life during treatment can reduce stigmatization of children with cancer and facilitate rehabilitation following treatment. The intervention program will be active in the periods during treatment when the child is attending their regular school for less than 3 days a week, 4 hours per day.

#### ***Physical and social intervention***

The supervised hospital-based physical and social activity program from diagnosis and throughout treatment includes daily participation in an individual training scheme and participation in joint physical and social activities twice a week during hospitalization. As the literature shows [23], we cannot reliably quantify the effect of a home-based training program, therefore the program is intended for physical activity in a hospital setting. The physical activity intervention focuses on muscle strength, cardio-respiratory fitness, and balance. Individual training sessions take place 3 to 5 days a week and training sessions vary from 5-120 minutes per session, depending on the type of training and the general condition of the child. The twice-weekly group training includes all study patients and ambassadors at the hospital on that specific day.

### Primary endpoints

#### ***Educational intervention***

The primary endpoint of the educational intervention is the child’s level of school achievement one year after cessation of first-line cancer treatment. The school achievement includes the child’s level of education in language, reading and writing skills, and mathematics. It is measured on a five-point response scale (outstanding level of performance, high level of performance, satisfactory level of performance, needs improvement in level of performance, and unsatisfactory level of performance).

#### ***Physical and social intervention***

The primary endpoint of the physical and social intervention is the level of VO_2max_, determined during exercise testing on a cycle ergometer after the Godfrey protocol [39], one year after the cessation of first-line cancer treatment.

### Secondary endpoints

The secondary endpoints include both quality of life and physical performance. We hypothesize that the components in the intervention will enhance the children’s quality of life 6 months after diagnosis and one-year post treatment. Quality of life is assessed using validated questionnaires and interviews.

### Outcome measures

The effects of the intervention program are quantified using validated questionnaires, physical fitness tests, blood tests, and full body dual energy X-ray absorptiometry (DEXA scan).

This comprehensive monitoring takes place at diagnosis, 3 and 6 months from baseline, one year after the cessation of treatment, and the long term effects of the program will continue to be monitored every 5 years.

### Questionnaires

***The PedsQL Core*** [40] measures the quality of life in children using 23 questions on a five-point response scale from never to almost always. The answers are divided into four domains: health and physical activity, emotions, dealing with others, and school activity.

***The Strength and Difficulties Questionnaire (SDQ)*** [41] consists of two parts. Part one includes 25 questions on a three-point response scale from does not fit to fits well in the following five areas: emotional symptoms, behavior, hyperactivity and concentration problems, problems with peers, and pro-social behavior. If the respondent confirms problems with concentration, behavior, or interaction with others, then part two of the questionnaire examines the duration, severity, extent, and social impact on the environment using a four-point response scale, from not at all to very much.

***The Revised Child Anxiety and Depression Scale (RCADS)*** [42] measures anxiety and depression using 47 questions on a four-point response scale from not at all to very often, according to the DSM-IV criteria. The RCADS includes the following subscales: separation anxiety, social phobia, generalized anxiety disorder, panic disorder, obsessive thoughts and actions, and depression.

***Resilience*** [43] measures resilience as personal competence, social competence, ability to maintain structure, family cohesion, and social support based on 28 questions on a five-point response scale from strongly agree to strongly disagree.

***The Loneliness and social Dissatisfaction Questionnaire (LDQ)*** [44] includes 24 questions to assess loneliness, social dissatisfaction, and hobbies on a three-point response scale with the answer categories no, sometimes, and yes.

***The Children’s Impact of Event Scale 13*** [45] measures problems which the child or adolescent may experience after a stressful event using 13 questions on a four-point response scale from not at all to often. It contains three subscales: intrusion, avoidance, and arousal.

***The best friend nominations scale*** [46] asks the child with cancer and his or her classmates to nominate their two best friends in the class. This provides a standardized total score for the number of nominations that each child receives and the mutual friendship score shows how many of their friendship choices are reciprocated. This is examined at the time of diagnosis and one year after treatment. The best friend nomination scale is disguised as a friendly exercise in the class, and not directly related to the child with cancer.

### Self-generated questionnaires

Self-generated questionnaires are used to record demographics, school participation, ambassador participation, the thoughts and reflections of schoolteachers at the time of diagnosis, evaluation of the educational sessions in class, academic position statements from teachers, physical activity before diagnosis, and physical training at home.

### Physical tests

The effect of the physical training program is assessed based on physical strength, balance, and a fitness test. The Andersen test [47] measures fitness using a 10-minute run or other high energy moving activity in intervals of 15 seconds of activity and 15 seconds of rest. The distance after 10 minutes and maximum and average heart rate is measured. The VO_2max_ is measured during a cycle ergometer test with the workload increasing progressively to the point of exhaustion [48] using a Hans Rudolph valve (2-way NRBV, Hans Rudolph Inc., Kansas City, MO, USA). The timed Up-and-Go test [49] tests basic mobility, defined as the ability to get in and out of bed, to get up and down from a chair, to walk short distances, and to turn. The test measures the time it takes to get up from a chair with armrests, walk 3 meters, turn, walk back to the chair and sit down again. The sit-to-stand test [50] evaluates muscle strength in the lower extremities and in the hip and core muscles. The test measures the number of repetitions of getting up from a sitting position to standing fully upright and sitting down again completed in 30 seconds. Hand strength as an indication of manual force is measured using a handheld dynamometer [51]. Two trials are conducted for each arm and are performed standing or sitting but with the elbow and dynamometer not touching anything. The flamingo balance test [52] measures the ability to balance on one leg and provides information about leg, hip, and abdominal muscle strength. The child is barefoot and balances on their preferred leg with the opposite leg lifted from the ground. The number of times the child loses balance in 60 seconds is registered. Each time the child loses balance the clock is stopped and then restarted when the child is ready.

Children in the intervention group and children in the control group diagnosed and being treated for cancer at Odense University Hospital, Aarhus University Hospital, and Aalborg University Hospital in the period 2013-2015, perform the physical tests.

### Statistical considerations I

**Figure 1:**
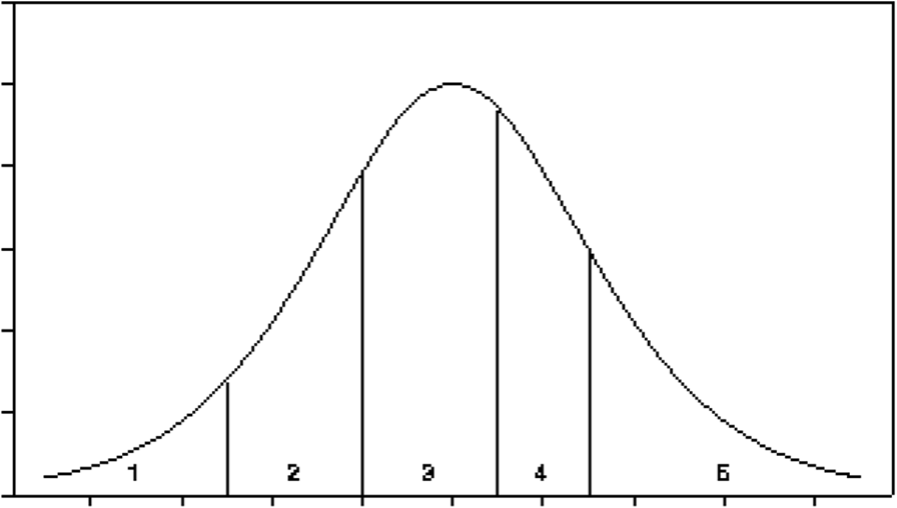
The structure of the ordinal regression model.

We expect all children aged 6-18 years diagnosed with cancer in Denmark during 2013-2015 will be included in the study. The primary endpoint for school achievement is classified using five ordinal categories: outstanding level of performance, high level of performance, satisfactory level of performance, needs improvement in level of performance, and unsatisfactory level of performance, with the frequency distribution of these five categories being 10%, 20%, 40%, 20%, and 10%, respectively (Figure 1). A change from one category to another is considered a significant change in the child’s school achievement (e.g. high level of performance to satisfactory level of performance). This endpoint will be analyzed using an ordinal regression model (details are given in Additional file 1) [53,54]. The primary endpoint of VO_2max_ in the intervention and control groups is determined by comparing the scores at diagnosis to the scores at one year after the cessation of treatment. This is done using an independent samples t-test. Additional analyses using the four study time points (diagnosis, 3 months after diagnosis, 6 months after diagnosis, and one year after cessation of treatment) will also be performed. Means and 95% confidence limits for the intervention and control groups will be computed at each time point using linear mixed models and the trajectories will be analyzed. A similar method will be used for the secondary endpoints but using only three time points for the questionnaire data. Linear mixed models will be used for each subscale of the PedsQL Core, the SDQ, the RCADS, Resilience, and the LDQ, and for the Andersen test, the timed Up-and-Go test, and the hand strength measurement. For the count data (the sit-to-stand test and the flamingo balance test), Poisson regression models with random effects will be used. Because the study is not randomized all comparisons of the two groups will be controlled for diagnosis. If any other variables are found to be unevenly distributed across the two groups additional analyses controlling for the effects of these variables will also be considered.

### Statistical considerations II and sample size calculations

This study has two primary endpoints therefore the type I error is set at 0.025 in the sample size calculations. For the primary endpoint in the educational intervention the ordinal regression model with a power of 0.90 outlined in Additional file 1 will to be able to detect a shift in the ordinal rating of approximately ½ a point, yielding a marginal frequency distribution for the five categories of 6%, 13%, 32%, 25%, 24%. Details are provided in Additional file 1.

The primary endpoint of the physical and social activity study is based on the power calculation derived from a pilot study [25] that found a baseline VO_2max_ of 24.3 ml/min/kg (SD 5.9) among children with acute lymphoblastic leukemia. If 120 children with cancer are included in both the intervention and control group, if the standard deviation of the change scores is 5.3 in both groups, and we use a significance level of 0.025, we will have a power of 0.90 to detect a 10% increase in the intervention group one year after cessation of treatment.

### Ethical approval

Information on the subjects is protected according to the Processing of Personal Data and Health Act. The Danish Data Protection Agency (file. 2007-58-0015/nr.30-0734) and the Regional Ethics Committee for the Capital Region (file. H 3-2012-105) approved the project, and the project complies with the Helsinki II declaration. In addition, the study is registered at Clinical Trials.gov (file. NCT01772849 and NCT01772862). Following oral and written information, parental, guardian and child’s (children above 15 year) written informed consent was obtained.

## Discussion

RESPECT is based on experience from an unpublished feasibility period, a theoretical framework, and earlier intervention studies of children with cancer, but reliable evidence on rehabilitation in children with cancer is lacking. RESPECT is the first nationwide study with sufficient power to reliably test the impact of a combined multimodal intervention program during treatment. The strength of this study is the combination of educational, psychosocial, and physical components, and the study has a more interactionistic and comprehensive perspective on rehabilitation than most of the previous studies.

Although a randomized trial would be optimal from a scientific point of view, it is unrealistic because the psychological, social, and ethical aspects mean only some of the children at the same unit will have ambassadors.

Another methodological consideration is the intervention starting at diagnosis. Starting an intervention study during the course of very toxic and intensive treatment causes logistical challenges related to the disease and the side effects of the treatment. At the time of diagnosis, families are very vulnerable and stressed, and there is a potential risk of declining participation. However, the feasibility period suggested a participation rate above 90%. Furthermore, the focus on rehabilitation may ameliorate the psychosocial burden on the child. The inclusion of two ambassadors from the child’s school class is unique in childhood cancer rehabilitation, but also carries potential ethical concerns related to the emotional stress and school performance of the ambassadors. The feasibility period indicated that we will be able to allocate two suitable ambassadors to more than 95% of patients, and that they will be able to cope with the challenges linked to their participation. To supervise this group we have an ambassador counsel chaired by a senior child psychologist, who is not involved in the daily operation of the project. Furthermore, an ambassador manual describes the selection procedures, safety measures, and intervention possibilities, to optimize identification and psychosocial monitoring. Before being accepted as an ambassador, and after every visit to the hospital, the ambassadors are screened and have clear follow-up plans arranged. Any adverse events and complications are continuously monitored and recorded.

Although physical exercise may cause sore muscles and stress injuries, there are no reports on such injuries in the existing literature on physical training of children with cancer. Another consideration is the level of intensity to which we exercise and test the children with cancer, from diagnosis and during treatment. Studies among children with heart diseases show that exercise training from low to high intensity is feasible and tolerable for seriously ill children [55] and it may have a positive effect on side effects such as fatigue [56]. While the primary endpoint of the physical intervention is not well documented for this study group, it is the gold standard in healthy children and a common outcome measurement in older cancer patients. The physical activity is adjusted to the treatment intensity and duration as well as the general condition of the child with cancer. During the physical intervention we monitor and assess adverse events.

We believe that RESPECT will contribute vital knowledge to the treatment and rehabilitation of children with cancer as well as other children hospitalized with long-term illness.

## Conclusion

This nationwide intervention study will have the power to reliably test the impact of a comprehensive combined educational, physical, and social intervention program on the recovery and rehabilitation of childhood and adolescent patients with cancer. It is the first study to examine the effect of an early rehabilitation program including the involvement of healthy classmates as ambassadors to help reproduce a normal everyday life for children undergoing cancer treatment in hospital.

## Competing interests

The authors declare that they have no competing interests.

## Authors’ contributions

The author LA is scientific director of the CIRE rehabilitation program. KS is scientific director, and HBL is the main project coordinator of the RESPECT project. TT is responsible for the physical activity intervention together with LBO, LFT and BS. ASH is responsible for the educational intervention program together with VS and NH. SAM is responsible for the ambassador safety. HH and PSW are responsible for the recruitment and inclusion of participants in the West Danish control group. KVA, CH, CJ and MM are all members if the program development and scientific board of the RESPECT project. KBC are responsible for the statistical analysis. All authors read and approved the final manuscript.

## Pre-publication history

The pre-publication history for this paper can be accessed here:

http://www.biomedcentral.com/1471-2407/13/544/prepub

## Supplementary Material

Additional file 1 **The ordinal regression model used for analysis of the educational primary endpoint **[53,54]. Click here for file
