# Supplementary material for: Study protocol: rehabilitation including social and physical activity and education in children and teenagers with cancer (RESPECT)
Source: BMC Cancer. 2013 Nov 14;13:544. doi: 10.1186/1471-2407-13-544 (PMC3832686; doi:10.1186/1471-2407-13-544)
Supplement: Additional file 1 — The ordinal regression model used for analysis of the educational primary endpoint [53,54]. [file 1471-2407-13-544-S1.docx]

**Additional file 1: The ordinal regression model used for analysis of the educational primary endpoint.**

Several regression models for ordinal data exist, the most widely used being the proportional odds model [55]. For this study we choose the following model based on the general idea of Muthén [56]. Let $X_{it}$ denote the ordinal rating given to child *i* at time *t, where t =0,1* indicates baseline and follow-up, respectively*.* The ordinal regression model used assumes that the distribution of $X_{it}$ is given by the probabilities:

$${P(X}_{it}=1)=\Phi(u_{it}-b_{1})$$

$${P(X}_{it}=2)=\Phi\left( {u_{it}-b}_{2} \right)-\Phi({u_{it}-b}_{1})$$

$${P(X}_{it}=2)=\Phi\left( {u_{it}-b}_{3} \right)-\Phi({u_{it}-b}_{2})$$

$${P(X}_{it}=2)=\Phi\left( {u_{it}-b}_{4} \right)-\Phi({u_{it}-b}_{3})$$

$${P(X}_{it}=1)=1-\Phi(u_{it}-b_{4})$$

where$\Phi$ is the cumulative distribution function of the normal distribution, $u_{i}$ is a parameter describing person *i*, and $\left( b_{1},b_{2},b_{3},b_{4} \right)$ are parameters estimated from the data.

This is an ordinal regression model that arises from focusing on the cumulative distribution of the ordinal response variable. The structure is illustrated in Figure 1.

The regression model specifies that:

$$u_{it}=a_{i}+t\delta g(i)$$

where $g(i)$ specifies group membership (intervention or control group). Sample size determination was done by simulating data sets with this structure using $\delta=0$ in the control group and $\delta=0.4$ in the intervention group. This corresponded to a shift in the average ordinal rating of approximately 0.5.

**Figure 1 legend**: The structure of the ordinal regression model
